# Tomophobia, the phobic fear caused by an invasive medical procedure - an emerging anxiety disorder: a case report

**DOI:** 10.1186/1752-1947-3-131

**Published:** 2009-11-18

**Authors:** Markus Schmid, Robert C Wolf, Roland W Freudenmann, Carlos Schönfeldt-Lecuona

**Affiliations:** 1 Department of Psychiatry and Psychotherapy III, University of Ulm, Leimgrubenweg 12, 98075 Ulm, Germany

## Abstract

**Introduction:**

Tomophobia refers to fear or anxiety caused by forthcoming surgical procedures and/or medical interventions.

**Case presentation:**

We present the case of a 69-year-old Caucasian man who refused urgently indicated medical intervention because of severe tomophobia.

**Conclusion:**

Due to the rising number of surgical interventions in modern medicine, as well as the high number of unrecognised cases of tomophobia, this common but underdiagnosed anxiety disorder should be highlighted.

## Introduction

Phobia (Greek: phobos, fear) is defined as an irrational, intense and persistent sensation of fear in relation to specific situations, activities, objects or individuals. Phobic anxiety disorders are conceptualized as a diagnostic subclass within the anxiety disorders. One of the main symptoms of this disorder is the excessive and unreasonable desire to avoid a feared subject or situation. When the fear is beyond one's cognitive or emotional control, or when the fear interferes with daily life activities, an anxiety disorder can be diagnosed.

The Diagnostic and Statistical Manual of Mental Disorders, Fourth Edition (DSM-IV) differentiates three groups of phobic anxiety disorder: social phobia, specific phobia, and agoraphobia [1]. According to DSM-IV the essential feature of a specific phobia (formerly simple phobia) is a marked and persistent fear of clearly discernible, circumscribed objects or situations. Exposure to the phobic stimulus almost invariably provokes an immediate anxiety response, which may take the form of a situationally bound or predisposed panic attack. Individuals suffering phobias recognize that the fear is excessive or unreasonable. Most often the phobic situation is avoided or else endured with intense anxiety or distress. The avoidance, anxious anticipation or distress in response to the feared situation interferes significantly with the person's normal routine, occupational (or academic) functioning, or social activities or relationships. There may also be a marked distress about having the phobia.

**Figure 1:**
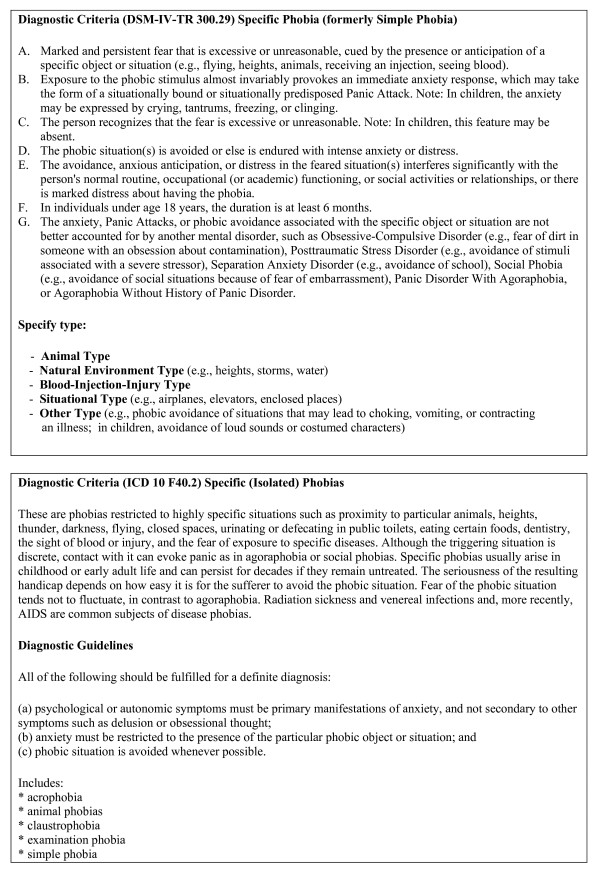
**This image shows the variant diagnostic criteria of DSM IV and ICD 10 **[1,8].

According to the Dresden Mental Health Study, the estimated life time prevalence of any specific phobia is 12.8% [2]. Within the concept of specific phobia the DSM-IV classification differentiates various subtypes that serve to indicate the focus of fear or avoidance, like "the animal type", "natural environment type", "blood-injection-injury type", "situational type" or "other type". In contrast, the International Classification of Disorders (ICD-10) does not considerer these different forms (Figure 1).

In many cases, patients present with more than one type of specific phobia (DSM-IV). The characteristics of the "blood-injection-injury" phobia include fear of seeing blood, becoming injured, or receiving an injection or other invasive medical procedure [3]. The highest prevalence of "blood-injection-injury" phobia is found in females in reproductive age (3.3%), while the prevalence in women over age 50 is 1.1%. Prevalence rates in men range from 0.7 to 0.8% [4]. We present the case of a 69-year-old patient who was diagnosed with tomophobia for the first time.

## Case presentation

The patient was a 69-year-old Caucasian man without a history of mental illness or any previous psychiatric treatment. He was initially transferred to a medical emergency department with marked dyspnoeic symptoms and tachycardia, where an acute coronary syndrome was diagnosed. After laboratory testing and an electrocardiogram a non-ST elevation myocardial infarction (NSTEMI) was diagnosed. The following coronary angiography (an intervention that was endured by the patient with enormous dread), revealed severe three-vessel disease.

The patient was informed of the urgent indication of a bypass operation, which was planned as an emergency intervention on the same day. At the end of the angiographic intervention, this information caused a severe panic reaction with hyperventilation, tachycardia and the feeling of loss of control, which was successfully treated with benzodiazepines. He described an intensely irrational and unavoidable fear of putting himself in the hands of others -surgeons and anaesthetists in this case. Moreover, the fear of losing control of his body through loss of consciousness or compromise of physical integrity during an operation or surgical intervention was reported. The patient was not able to give his agreement for the operative intervention because of overwhelming panic and anxiety. Due to his intense fear he eventually refused the bypass operation. This dramatic situation led to a condition of anxiety, strain and severe agitation, which led to a psychiatric referral and, consecutively, to an in-patient psychiatric admission.

The patient was relieved by the psychiatric admission and the understanding of his phobic fear. He reported a 20-year history of severe coxarthrosis, which caused serious pain and progressive leg deformation and malfunction, and which had never been operated on because of his fear of surgery. The clinical examination revealed that his left leg was 4.5 cm shorter than his right leg. He also had a marked impaired gait due to a limp in his left leg. Additionally, the coxarthrosis led to an extensive inguinal hernia due to pain induced mismovement. He also refused the necessary inguinal operation for over five years due to the same phobic symptoms. Further phobic symptoms and other symptoms of anxiety were explored, such as his fear about GP visits and discussions with superior colleagues. The patient reported an age of onset in the early adult years. With regard to childhood and adolescence, he described fear symptoms while knocking on or opening 'foreign' doors. Though the patient showed avoidant personality traits, no personality disorder could be diagnosed.

The psychopathological findings at the time of psychiatric exploration were limited to intense fear in relation to the forthcoming surgical procedures and interventions. During the psychiatric exploration, the patient was polite, friendly, and honest. Compulsive symptoms were limited to the repeated checking of electric appliances. As a consequence of his lifelong avoidance strategies he seemed not to feel oppressive limitations in everyday life. Until then, he had never consulted a psychiatrist or a psychotherapist regarding his phobic symptoms. He described being ashamed of his unreasonable fear symptoms. Panic disorder symptoms were not observed at any time during the psychiatric exploration. No history of syncope was found. Family history revealed a suspected anxiety disorder in the patient's father, although he reportedly never consulted any physician or other healthcare professional. Further examinations of the patient such as laboratory tests, duplex sonography, an electroencephalogram and a cranial magnetic resonance imaging were entirely normal. A Specific Phobia was diagnosed according to DSM-IV criteria.

To improve the intense fear reported by the patient when being confronted with the problem of the necessity of the operation, a psychotropic treatment with escitalopram 10 mg once a day and pregabalin 150 mg twice a day was initiated. The patient described an improvement of these fear symptoms. Supported by intensive conversational therapy based on cognitive behavioural techniques, he stabilized and was subsequently discharged. The patient still refused the invasive procedure, as his fear of the procedure continued to overwhelm his fear of dying from a heart attack. Behavioural psychotherapy as an out-patient treatment was recommended in order to diminish the patient's phobic fears.

## Discussion

Our patient suffered from a specific phobia "blood-injection-injury type", in this particular case provoked by medical interventions including the forthcoming coronary bypass operation. This specific phobia is also termed "tomophobia" (Greek: tomos, cut). According to related studies, "blood-injection-injury" phobia is characterized by combined fear and disgust responses [5]. A frequent symptom of "blood-injection-injury" phobia that distinguishes it from other specific phobia subtypes is syncope [3].

Tomophobia is sometimes accompanied by the irrational fear of dying under anaesthetics during a chirurgic intervention. Our patient neither experienced syncope nor symptoms of massive disgust while being confronted with the phobic stimuli, but he complained of intense fears related to the impending operation. Considering the absence of disgust response and fainting, the assignment to the situational subtype or a combined form of phobia could be the more appropriate diagnostic category for the reported case of tomophobia.

Bienvenu *et al*. reported a study of 1920 subjects, which showed a prevalence of the "blood-injection-injury" phobia of 3.5%. None of these patients was receiving mental health treatment specifically for phobia [6]. With regard to tomophobia, the number of undiagnosed cases might be much higher than the number of cases that are actually diagnosed, possibly because repression and avoidance of feared situations are the leading behaviour of these phobic patients. The majority of patients suffering from specific phobia do not seek professional psychiatric or psychotherapeutic help (only 12-30% do) unless they have a comorbid disorder [1]. In addition, the presence of "blood-injection-injury" related symptoms worsen the prognosis of panic disorder and agoraphobia [7].

Due to progress in the development of invasive treatment and an increased number of established intervention procedures in modern medicine, cases of diagnosed tomophobia might increase in the near future. Above all, surgeons and general physicians may be increasingly confronted with patients who refuse medically urgent procedures due to tomophobic fears. Our patient became symptomatic when he was informed about the indication of the necessary operation. The patient's refusal of the surgical intervention can be comprehended as typical avoidance behaviour as a result of his permanent phobic disorder. The patient was always cognitively capable of understanding the consequences of his unreasonable decision, but the fear of impairment of physical integrity and of losing control while accepting the bypass operation was greater than the fear of dying as a consequence of the detected heart disease.

## Conclusion

We present the case of a patient who refused recommended urgent coronary bypass surgery because of tomophobic fears. Due to the rapid progress of modern medicine, including frequent use of invasive medical procedures, tomophobia will likely be an increasingly common and clinically impairing anxiety disorder. We assume that tomophobia is often unrecognised, which consequently limits its diagnosis and treatment. With this case report we would like to highlight a common but still largely unappreciated anxiety disorder and encourage improved diagnosis and treatment of patients suffering from it.

## Abbreviations

DSM IV: The Diagnostic and Statistical Manual of Mental Disorders, Fourth Edition (DSM-IV); ICD 10: International Classification of Disorders; NSTEMI: non-ST elevation myocardial infarction.

## Consent

Written informed consent was obtained from the patient for publication of this case report and any accompanying images. A copy of the written consent is available for review by the Editor-in-Chief of this journal.

## Competing interests

The authors declare that they have no competing interests.

## Authors' contributions

MS and CS admitted the patient and were responsible for his treatment. They also contributed in writing the manuscript, together with RC and RF. All authors read and approved the final manuscript.

## References

[B1] Diagnostic and statistical manual of mental disorders 2000 4 Washington, DC; American Psychiatric Association

[B2] Becker ES Rinck M Türke V Kause P Goodwin R Neumer S Margraf J Epidemiology of specific phobia subtypes: findings from the Dresden Mental Health Study Eur Psychiatry 2007 22 2 69 74 10.1016/j.eurpsy.2006.09.006 17157482

[B3] Gerlach AL Spellmeyer G Vögele C Huster R Stevens S Hetzel G Deckert J Blood-injury phobia with and without a history of fainting: disgust sensitivity does not explain the fainting response Psychosomatic Medicine 2006 68 331 339 10.1097/01.psy.0000203284.53066.4b 16554401

[B4] Bracha HS Bienvenu OJ Eatond WW Testing the Paleolithic-human-warfare hypothesis of blood-injection phobia in the Baltimore ECA Follow-up Study. Towards a more etiologically-based conceptualization for DSM-V J Affect Disord 2007 97 1 4 10.1016/j.jad.2006.06.014 16860872

[B5] Koch MD O'Neill HK Sawchuk CN Connolly K Domain-specific and generalized disgust sensitivity in blood-injection-injury phobia: the application of behavioural approach/avoidance tasks J Anxiety Disord 2002 16 5 511 527 10.1016/S0887-6185(02)00170-6 12396209

[B6] Bienvenu OJ Eaton WW The epidemiology of blood-injection-injury phobia Psychol Med 1998 28 1129 1136 10.1017/S0033291798007144 9794020

[B7] Overbeek T Büchold H Schruers K Griez E Blood-injury phobic avoidance as predictor of nonresponse to pharmacotherapy in panic disorder with agoraphobia J Affect Disord 2004 78 227 233 10.1016/S0165-0327(02)00312-9 15013247

[B8] World Health Organisation The International Classification of Disorders, 10th Revision, (ICD-10) 1991 Bern, Huber

